# A CAGED DEATH: EFFORTS TO IMPROVE QUALITY OF LIFE AND DEATH FOR AGING INCARCERATED WOMEN

**DOI:** 10.1093/geroni/igae098.3196

**Published:** 2024-12-31

**Authors:** Tosha Big Eagle, L Shatswell, Kelly O’Sullivan, Raven Weaver, Cory Bolkan

**Affiliations:** Washington State University, Vancouver, Washington, United States; University of Puget Sound, Tacoma, Washington, United States; Washington State University, Pullman, Washington, United States; Washington State University, Pullman, Washington, United States; Washington State University, Pullman, Washington, United States

## Abstract

Older incarcerated women are overlooked in research despite facing significant challenges; many remain incarcerated until death, even though prisons are not designed to provide sufficient geriatric or end-of-life care. This scoping review investigated the intersectionality between aging, incarceration, and health outcomes, focusing on older women, particularly those from historically marginalized social identities. With limited research on the effects of incarceration, especially for older, minoritized populations, we adopted a comprehensive approach, spanning between 1990 - 2023 and utilizing Academic Search Complete, APA PsycINFO, CINAHL, and PubMed databases. The search employed tiered keywords related to incarceration, jail/prison; aging, older adults; support, services, health; and women, race/ethnicity, culture. Two independent reviewers screened and identified 85 relevant articles for detailed analyses. Findings suggest that health outcomes, including disruption to physical, cognitive, and psychosocial development, are exacerbated among older women. Further, women ‘s prisons lack peer caregiving programs available in some men’s prisons, highlighting gender inequities. Results underscore the critical role of adequate healthcare access to improve quality of life and death during incarceration, and successful re-entry into society, when applicable. There is also a dire need for gender-specific care, preventative healthcare, and early intervention strategies to address the unique health needs of incarcerated women as they age and die in prison. Few examples of programs/policies providing equitable access to comprehensive healthcare services are available. Future research contextualizing the intersectionality of aging, incarceration, and health outcomes, can contribute to this underexplored field, urging an inclusive and holistic approach to the well-being of incarcerated older women.

